# Iron Deficiency Anaemia in Pregnancy and Postpartum: Pathophysiology and Effect of Oral versus Intravenous Iron Therapy

**DOI:** 10.1155/2012/630519

**Published:** 2012-06-26

**Authors:** Alhossain A. Khalafallah, Amanda E. Dennis

**Affiliations:** ^1^Department of Haematology, Launceston General Hospital, Launceston, Tasmania 7250, Australia; ^2^School of Human Life Sciences, University of Tasmania, Australia; ^3^Department of Obstetrics and Gynaecology, Launceston General Hospital, Tasmania 7250, Australia

## Abstract

Nutritional iron-deficiency anaemia (IDA) is the most common disorder in the world, affecting more than two billion people. The World Health Organization's global database on anaemia has estimated a prevalence of 14% based on a regression-based analysis. Recent data show that the prevalence of IDA in pregnant women in industrialized countries is 17.4% while the incidence of IDA in developing countries increases significantly up to 56%. Although oral iron supplementation is widely used for the treatment of IDA, not all patients respond adequately to oral iron therapy. This is due to several factors including the side effects of oral iron which lead to poor compliance and lack of efficacy. The side effects, predominantly gastrointestinal discomfort, occur in a large cohort of patients taking oral iron preparations. Previously, the use of intravenous iron had been associated with undesirable and sometimes serious side effects and therefore was underutilised. However, in recent years, new type II and III iron complexes have been developed, which offer better compliance and toleration as well as high efficacy with a good safety profile. In summary, intravenous iron can be used safely for a rapid repletion of iron stores and correction of anaemia during and after pregnancy.

## 1. Iron Deficiency in Women 

Nutritional iron deficiency is the most common deficiency disorder in the world, affecting more than two billion people worldwide, with pregnant women at particular risk [1–3]. World Health Organization (WHO) data show that iron deficiency anaemia (IDA) in pregnancy is a significant problem throughout the world with a prevalence ranging from an average of 14% of pregnant women in industrialized countries to an average of 56% (range 35–75%) in developing countries [2, 3].

Furthermore, IDA not only affects a large number of women and children in the developing world, but is also considered the only nutrient deficiency that is significantly prevalent in the developed world also. The number of patients with ID and IDA is overwhelming as more than 2 billion people, approximately 30% of the world's population, are iron deficient with variable prevalence, distribution, and contributing factors in different parts of the world [1–3].

Iron deficiency affects more women than any other condition, constituting an epidemic public health crisis. It is usually present with subtle manifestations and should be considered as a chronic slowly progressing disease that is often underestimated and untreated worldwide despite several warnings and awareness campaigned by the WHO [1–3].

The high prevalence of IDA in women has substantial health consequences with subsequent socioeconomic hazards, including poor pregnancy outcome, impaired educational performance, and decreased work capacity and productivity [1, 4].

Because of the magnitude and consequences of iron deficiency anaemia in the world, especially in women in their childbearing period, several international conferences on nutrition have addressed this issue in order to reduce the prevalence of iron deficiency in women of childbearing age without major success [1–6]. The consequences of IDA have been widely studied [7–10]. However, there remains a lack of data about its effects on patient's wellbeing.

Targeted iron supplementation, an iron-rich diet, or both, can improve iron deficiency. However, the variability of bioavailable iron compounds limits its value against nutritional iron deficiency. Therefore, laboratory measures of iron stores should be utilised to determine iron deficiency and monitor therapy [3–6].

This review highlights the importance of IDA in pregnancy and discusses appropriate treatment in order to avoid serious complications of anaemia.

## 2. Iron Metabolism 

The balance of iron metabolism in healthy individuals predominantly reflects three variables: nutritional intake, iron loss, and current demand. The nutritional iron intake relates to the amount of digested iron in food and the ability to absorb iron from the digestive tract [4]. The amount of iron absorbed depends largely on the presence or absence of pathology of the gastrointestinal tract or a comorbidity (such as chronic inflammatory diseases) that may result in expression of the iron regulatory proteins and a peptide called hepcidin, which ultimately blocks iron absorption [11–13].

The main source of iron in humans comes from the destruction of erythrocytes by macrophages of the reticuloendothelial system including the spleen or in other words, a recycled internal iron supply. Recent studies have shown how the human body up- and downregulates iron absorption in response to changing iron status via intestinal and hepatic proteins [12–15].

### 2.1. Iron Metabolism in Pregnancy

During pregnancy, fetal hepcidin controls the placental transfer of iron from maternal plasma to the fetal circulation. When hepcidin concentrations are low, iron enters blood plasma at a high rate. When hepcidin concentrations are high, ferroportin is internalized, and iron is trapped in enterocytes, macrophages, and hepatocytes [11, 15]. The daily requirement of external iron remains as little as between 1 to 8 mg daily [16]. However, more external iron is required to balance increased demand for iron especially with physiological requirements during growth, pregnancy, and lactation [16, 17]. This significant increased demand for iron is required to develop the fetus and placenta in addition to support mother's blood volume. Furthermore, pregnant women are subject to iron loss during and after delivery [16–18].

The total iron loss associated with pregnancy and lactation is approximately 1000 mg [16, 17]. Therefore the recommended daily dietary allowance for iron in pregnancy is 27 mg instead of 8 mg in the adult nonpregnant population. Lactation requires a daily dietary allowance of 10 mg. [16–18].

## 3. Laboratory Markers for Iron Status

### 3.1. Definition of Anaemia in IDA in Pregnant and Nonpregnant Women

Anaemia of pregnancy is generally defined as Hb <110 g/L or <115 g/L in some clinical practice guidelines with a slight variation according to the trimester of pregnancy. However, a haemoglobin level <100 g/L indicates anaemia at any stage during pregnancy that should initiate investigations and treatment because of potentially serious consequences for the mother and her baby, with an increased risk of intrauterine growth retardation and premature birth. In the meantime, anaemia in women of reproductive age is defined as Hb <120 g/L or in some studies <115 g/L as this is laboratory and population specific [7–10].

### 3.2. Definition of Iron Deficiency (ID)

Iron deficiency can be classified as severe ID when the serum ferritin level is below 20–30 *μ*g/L and mild-moderate ID if the serum ferritin level is below 70–100 *μ*g/L. Ferritin level is considered the surrogate marker for ID. However, serum ferritin is an acute phase reactant and may be raised in cases of inflammation or infection, therefore a concurrent test for inflammatory markers is advisable in cases of anaemia with raised ferritin to exclude reactive causes. ID is most likely not present if the ferritin level is above 100 *μ*g/L [10].

Although a study of bone marrow iron stores is generally regarded as the definitive marker of iron deficiency, it remains an impractical and invasive procedure to apply for most patients. Measurement of both soluble transferrin receptor and serum ferritin provides a tool for accurate diagnosis of IDA [19–21]. However, transferrin receptor is not a well-standardized test that can be reliably reproduced with high precision in most laboratories worldwide [21].

In the meantime, ferritin estimation is an easy automated test to perform in most laboratories in the world; however, its use is limited in cases of inflammation or infection as it is considered to be influenced by acute phase responses and hence negatively influences its value in clinical interpretation of the test results [19, 20]. The commonly available laboratory tests that determine iron status, namely, serum iron, transferrin, total iron-binding capacity (TIBC), transferrin saturation, and ferritin are widely used in worldwide clinical practice [19, 20].

Soluble TfR (sTfR) is present in human plasma and is considered as a truncated form of the tissue receptor that exists as a transferrin-receptor complex and therefore it reflects tissue iron deficiency [21]. Another protein that plays a crucial role in iron metabolism is hepcidin, which is primarily made by hepatocytes and secreted into the blood circulation. Hepcidin is a small-sized molecule composed of 25-amino acid peptide, which is renally excreted and therefore can be detected and measured in urine [14, 15]. Furthermore, hepcidins rapid excretion suggests that it is regulation triggered at the level of production sites. Hepcidin circulates in the ferroportins plasma and responds to various stimuli that regulate iron stores and serum iron [15].

Recent studies demonstrate that hepcidin levels are reduced in iron deficiency [14, 15]. Measurement of blood or urine hepcidin levels can be achieved by mass spectrometry and immunoassays in serum, plasma, and urine [22]. However, the diagnostic utility of serum hepcidin in iron deficiency has not yet been defined in clinical application [23]. Nevertheless, hepcidin estimation seems a potentially accurate test that reflects the actual iron status with less limitations.

Altogether, new technology such as hypochromic reticulocytes and reticulocyte haemoglobin testing, sTfR, and hepcidin have reportedly been developed with higher sensitivity, specificity, reproducibility, and cost effectiveness [19–24]. This may offer a reliable screening tool for iron deficiency in the future. It is worth noting that there are no specific data addressing the difference of these markers in the pregnant versus nonpregnant population. However, in principle, no essential change should occur in iron metabolism in the pregnant versus non-pregnant population except for the increased iron demand as discussed before.

### 3.3. Current Strategy to Assess Iron Deficiency during Pregnancy

Full blood count and MCV value allowing the diagnosis of microcytic anaemia is considered a good screening tool for IDA. However, in areas of the world where haemoglobinopathies are prevalent and these may be associated with microcytosis, iron studies, in particular ferritin level remains the surrogate marker for IDA. According to the ferritin level, iron deficiency can be classified as severe ID when the ferritin level is <30 *μ*g/L or mild-moderate ID if ferritin <100 *μ*g/L and >30 *μ*g/L (there is a wide normal range between 20 and 464 and is laboratory and method specific) [8]. In cases of elevated ferritin >100 *μ*g/L with a concurrent anaemia, a reactive common cause such as infection should be excluded and other causes of anaemia should be examined accordingly. Other complementary tests in iron studies such as serum iron, iron binding capacity, and transferrin saturation are helpful in confirming the diagnosis of IDA.

## 4. Oral versus Intravenous Iron for Treatment**** of Iron Deficiency in Women of Reproductive Age and Pregnancy

Oral iron therapy is the most widely prescribed treatment for iron deficiency anaemia, however, there are many issues that may prevent oral iron supplementation from successfully managing IDA. For instance, many patients do not respond adequately to oral iron therapy due to difficulties associated with ingestion of the tablets and their side effects. Side effects may play a significant role in rates of compliance [25, 26]. Furthermore, the presence of bowel disease may affect the absorption of iron and thereby minimize the benefit received from oral iron therapy [27–29].

The side effects of oral iron therapy include gastrointestinal disturbances characterized by colicky pain, nausea, vomiting, diarrhoea, and/or constipation, and occur in about 50% of patients taking iron preparations [13, 27–29].

Furthermore, the most widely prescribed oral iron is mainly composed of ferrous salts [25–27]. Ferrous salt is characterized by low and variable absorption rates. Its absorption can be limited by ingestion of certain foods as well as mucosal luminal damage [27–29]. Therefore, ferric compounds were introduced to avoid such obstacles. However, these compounds are generally less soluble and have poor bioavailability [29].

The usual recommended oral iron sulphate dose for the treatment of iron deficiency is at least 80 mg daily of elemental iron, which is equivalent to 250 mg of oral iron sulphate tablets (Abbott, Australasia Pty Ltd.).

Iron absorption requires an acidic medium, therefore its absorption may be decreased by intake of antacids or proton pump inhibitors and histamine receptor antagonists. Interference of iron absorption may occur with the intake of certain medications, which thereby minimises the benefit received from oral iron treatment [29].

The major challenges in the management of IDA are related to the tolerability and side effects of iron therapy in its different forms. Therefore, it is crucial to determine the most appropriate form and dose of iron as well as duration of treatment in order to successfully replenish iron stores. Although oral iron is widely used worldwide, the effectiveness of oral iron is largely compromised by lack of absorption, poor compliance, increased adverse effects (up to 56%), and discontinuation of treatment (up to 20%) [4, 26, 29].

Therefore, parenteral iron is seen to be an attractive option in the treatment of IDA and is likely to be more popular due to the introduction of new intravenous iron preparations, which allow high doses of iron to be administered rapidly in a single treatment [30–32].

### 4.1. Side Effects of IV Iron

In the past, intravenous iron had been associated with undesirable and sometimes serious side effects and was therefore limited in use [33, 34]. However, in recent years, new type II and III iron complexes have been developed which are better tolerated and can be used for rapid repletion of iron stores [34, 35]. Despite the increasing evidence of the safety of the newer preparations, both in pregnant and general populations, intravenous iron continues to be underutilised because of previous concerns with tolerability of older intravenous iron preparations [7, 8, 30].

Review of infusions of iron dextran among 481 patients of both sexes revealed that about 25% of patients had mild side effects, which were self-limiting. However, about 2% experienced severe allergic reactions and about 0.6% were considered as anaphylactic reactions. Most of these reactions occurred immediately during the infusion of the test dose [36].

Iron gluconate is considered to have a lower reaction rate and therefore a test dose is not recommended with only 3.3 allergic events per million doses per year with iron gluconate reported [37]. There were no life-threatening reactions recorded as a result of iron gluconate infusion. On the other hand, there were 31 fatalities among 196 allergic/anaphylactic reactions, which were reported for iron dextran [37].

The high incidence of adverse reactions to iron dextran, including serious adverse events have limited its application in pregnancy [38–40, 43]. Whilst the application of iron gluconate is considered safe, it remains impractical in theory as it requires multiple infusions with huge implications on the often limited health system resources as well as on patients' compliance.

More recently new forms of intravenous iron that have been developed and are available, are permitting treating physicians to safely administer relatively high doses of iron in a single dose treatment.

### 4.2. Intravenous versus Oral Iron Therapy in Pregnancy

Intravenous iron, including iron sucrose, was employed in randomised controlled trials with improved effectiveness of intravenous iron only or in combination with oral iron, compared to oral iron only, based on Hb levels [41–43].

A single IV iron sucrose dose has been reported to be associated with an increased incidence of thrombosis (9/41, 22%) [43]. In contrast, 6 small doses of intravenous iron sucrose administered over a three-week period were without infusion-associated thrombosis, with intravenous iron sucrose administered in 5 daily doses to 45 pregnant women, also well tolerated [41]. In the first study utilising intravenous iron sucrose, there was no significant difference in Hb levels at any time measured at days 8, 15, 21, and 30 and at delivery [42] between intravenous iron sucrose or oral iron sulphate. In contrast, in another trial, with 6 small doses of iron sucrose, there was a significant difference in Hb levels in favour of the intravenous iron sucrose group as measured at 2 and 4 weeks after administration of IV iron and at delivery [41]. However, both trials administered IV iron sucrose at the expense of a vastly greater effort from the patients to present to the hospital for 6 infusions in a short period of time as well as the extra demands on hospital resources [41, 42].

Furthermore, relatively older and established iron preparations such as intravenous iron polymaltose (Ferrosig, Sigma Pharmaceuticals, Australia) demonstrated a high safety profile in the treatment of IDA in both obstetric and general populations without a maximum dose of treatment [30]. The total dose of IV iron polymaltose is calculated according to the patient's body weight and entry Hb level with reference to the product guidelines as follows: iron dose in mg (50 mg per 1 mL) = body weight in kg (maximum 90) × target Hb (120 g/L) − actual Hb in g/L × constant factor (0.24) + iron depot (500). Iron polymaltose infusion showed high efficacy and safety profile during pregnancy in the largest, recently published trial [30].

In this study, two hundred Caucasian pregnant women aged 18 years or above were identified with moderate IDA, defined as Hb ≤115 g/L (reference range (RR) 120–160 g/L) and low iron stores based on a serum ferritin level <30 *μ*g/L (RR 30–440 *μ*g/L). The IV arm required a single intravenous infusion of iron polymaltose (Ferrosig, Sigma Pharmaceuticals, Australia) within 1 week after antennal clinic booking, usually after 12 weeks of gestation, followed by oral maintenance therapy. IV iron was commenced during the 2nd and 3rd trimesters only. The oral treatment arm comprised iron sulphate 250 mg tablets (elemental iron 80 mg, Abbott, Australasia Pty Ltd.) to be taken daily within two days after booking until delivery [30]. At preenrolment, there were no significant differences in the dietary iron intake or supplement intake between the two groups based on a specially designed questionnaire addressing these issues. The participants were followed up during the pregnancy and at a postdelivery median follow-up period of 32 months (range 26–42). Iron status and haemoglobin were determined at time of entry in the study as a baseline, then prior to delivery and thereafter 4 weeks after delivery [30].

As reported in the original study, at delivery the proportion of women with lower than normal ferritin levels was 79% for women who were treated with oral iron as compared to 4.5% for women who received IV iron (*P* < 0.001) [30]. The percentage of women at delivery with Hb level <116 g/L was 29% in the oral iron group versus 16% in the IV iron group (*P* = 0.04) [30].

As a common practice at our institution, we have performed more than 1000 IV iron polymaltose infusions for the treatment of IDA in pregnancy during the last 5 years. Most of the women tolerated the IV iron polymaltose well without major side effects. There was no recorded anaphylaxis or mortality secondary to IV iron in this cohort of patients.

In unpublished data collected as a follow-up study of the original trial [30], there was a significant improvement in the general health of women who received IV iron polymaltose versus oral iron (*P* < 0.001). The duration of breast feeding was longer (*P* = 0.04) in those women who had received IV iron polymaltose versus oral iron. Women with better iron status were less downhearted (*P* = 0.005) and less likely to develop postnatal clinical depression (*P* = 0.003).

This would indicate that it is worthwhile considering the Hb and iron status as a surrogate marker for assessment of women's wellbeing, not only during pregnancy but also during the postnatal period. However, further studies are warranted to confirm and extend these findings.

Furthermore, recent reports demonstrate the feasibility of rapid iron polymaltose infusion over 2 hours [30, 44, 45]. However, a test dose of iron polymaltose (100 mg) should be first administered over 30 minutes, and premedication with antihistamine and/or low-dose steroids is recommended prior to iron treatment for better toleration [44, 45].

A recent comprehensive meta-analysis and review by Reveiz et al. [7] of the literature between 1970 till present on different treatments for IDA of pregnancy showed paucity of good quality trials assessing clinical maternal and neonatal effects of iron administration in women with IDA in spite of the high incidence and burden of disease associated with IDA. During this period, there was only one prospective randomized trial of the effect of IV iron versus oral iron in the treatment of IDA during pregnancy that fulfils the stringent independent reviewer quality criteria [7, 30].

## 5. Recent Data on Treatment of IDA in the ****Postpartum Period

The new preparations of intravenous iron (Table 1) are seeking approval for use during pregnancy in phase II and III clinical trials from the authorised organisational bodies in Europe and the USA. Nevertheless, they can be potentially used currently in the non-pregnant female population for the treatment of postpartum, pre-further, and postmenopausal iron deficiency anaemia according to the regional health authority approval.

In a randomised trial to assess safety and efficacy of intravenous ferric carboxymaltose in the treatment of postpartum IDA, 227 women were assigned to IV ferric carboxymaltose with 1000 mg maximum dose (up to 3 weekly doses) versus 117 women who received oral ferrous sulphate 100 mg twice daily [52]. Intravenous iron carboxymaltose was as effective as oral ferrous sulfate with no statistically significant differences between groups at any time point despite the shorter treatment period and a lower total dose of iron (mean 1.3 g IV iron versus 16.8 g oral iron). Furthermore, in the IV iron carboxymaltose group, the increases in ferritin levels were significantly greater than in the ferrous sulphate (*P* < 0.0001) indicating a successful repletion of iron stores and accessibility for erythropoiesis [52].

In a multicenter randomized, controlled study, 291 women directly after delivery with haemoglobin ≤100 g/L were randomized to receive 1000 mg IV iron carboxymaltose (143 women), repeated weekly to a calculated replacement dose (maximum dose 2.5 g), or ferrous sulfate (148 women) 325 mg orally three times daily for 6 weeks (total dose 40.9 g) [53]. Ferric carboxymaltose-treated women achieved a haemoglobin >120 g/L in a shorter period of time with a sustained haemoglobin >120 g/L at day 42. Furthermore, the achieved haemoglobin rise of ≥30 g/L was significantly more rapid in the IV iron group than the oral group in achieving higher serum ferritin levels. Drug-related adverse events occurred less frequently with ferric carboxymaltose [53].

In a phase 3 randomised trial 174 women who received IV ferric carboxymaltose with a mean total dose of 1.4 g versus 178 women who received 325 mg ferrous sulfate three times daily for 6 weeks (total dose 40.9 g) were assessed [54]. Patients assigned to IV ferric carboxymaltose achieved a haemoglobin rise >20 g/L faster than the oral iron group (7 days compared with 14 days in the oral iron group, *P* < 0.001). The IV iron group significantly achieved a haemoglobin rise >30 g/L at any time (86.3% compared with 60.4% in the oral iron group, *P* < 0.001), and were more likely to achieve a haemoglobin >120 g/L (90.5% compared with 68.6%, *P* < 0.001). In the meantime, there were no serious adverse drug reactions in both groups [54].

In a large randomized, controlled phase 3 multicentre trial, 477 women with IDA and heavy uterine bleeding were assigned to receive either IV ferric carboxymaltose (230 women) with a maximum dose of 1000 mg repeated weekly to achieve a total calculated replacement dose, or 325 mg of oral ferrous sulphate (65 mg elemental iron) three times daily for 6 weeks with a total dose of 40.9 g in 226 women [55]. Twenty-one patients did not receive the assigned treatment in this study.

About 82% of the IV iron arm achieved haemoglobin rise ≥20 g/L versus 62% in the oral iron *P* < 0.001. Women who achieved a haemoglobin rise ≥30 g/L were 53% in the IV iron group versus 36% in the oral iron group (*P* < 0.001). Also, more women (73%) achieved normal haemoglobin >120 g/L in the IV iron group compared to 50% in the oral iron group (*P* < 0.001). There were no serious adverse drug events. This trial demonstrated that patients with IDA due to heavy uterine bleeding who received IV iron carboxymaltose, are more likely to have normal haemoglobin with replenished iron stores [55].

Altogether, the new intravenous iron preparations represent a medical revolution in effective, rapid, and safe iron repletion in the management of iron deficiency anaemia [46–55]. This will positively reflect on the treatment of IDA in different populations by application of a single high-dose intravenous iron treatment with effective subsequent repletion of iron stores and hence improvement of subjective and objective outcomes of the IDA. Although iron deficiency is a precursor of IDA, many clinical studies treat it similarly to IDA.

## 6. Cost Effectiveness 

The cost of one iron sulphate tablet is approximately USD $0.3, so the average cost throughout one pregnancy is calculated to be between $54 and $89. The cost of iron polymaltose containing 500 mg is $50, so the average treatment cost is $100. In Australia, the cost of the outpatient hospital visit and nursing time for the IV iron adds approximately $60–$100 to the drug cost subject to variations according to different health systems. The cost of the new iron preparation ferric carboxymaltose is approximately $272 per average 1000 mg total dose compared to $280 for 1000 mg of iron sucrose (Table 2). This cost analysis is subject to change according to different health systems and countries.

## 7. Avoiding Blood Transfusion

In the case of severe IDA, a blood transfusion has been the traditional efficient approach to correct anaemia, especially if patients did not respond to oral iron therapy or when a rapid correction of anaemia is clinically required. Although there is a lack of data regarding the avoidance of blood transfusion during pregnancy, a recent trial investigating treatment of IDA with oral versus IV iron in pregnancy demonstrated that none of both treatment arm participants received blood transfusion for correction of anaemia during pregnancy. However two patients (0.9%) in the oral iron arm received blood transfusion in the postpartum period [30].

Currently, the development of new intravenous iron formulations that offer higher doses in a single administration has provided the treating physicians with the opportunity to employ intravenous iron as an effective, rapid, and safe treatment for IDA [46–55], avoiding the use of blood transfusion with its known hazards [56]. There is increasing evidence-based research that supports the safety and efficacy of IV iron in IDA. There is also increasing evidence for inadequacy of oral iron in terms of adverse effects, lack of compliance as well as lack of absorption and slow and often questionable effect in IDA patients [34, 35, 42].

A common requirement across the range of clinical situations is the need for safe, effective, higher, and less frequent doses to achieve optimal clinical outcomes. The major goals of such strategies include overall cost reduction, relief to overstretched health system(s), improved patient convenience, improved compliance, preservation of venous access, and reduced blood transfusion [34, 56, 57]. This will ultimately reduce the demand for blood transfusions, especially in the case of short supply. Furthermore, some of the new iron preparations such as ferric carboxymaltose and iron isomaltoside do not require a test dose and therefore, ease the application of intravenous iron in a timely and cost-effective fashion. This certainly will enhance the use of intravenous iron in clinical practice.

## 8. Summary 

**Figure 1:**
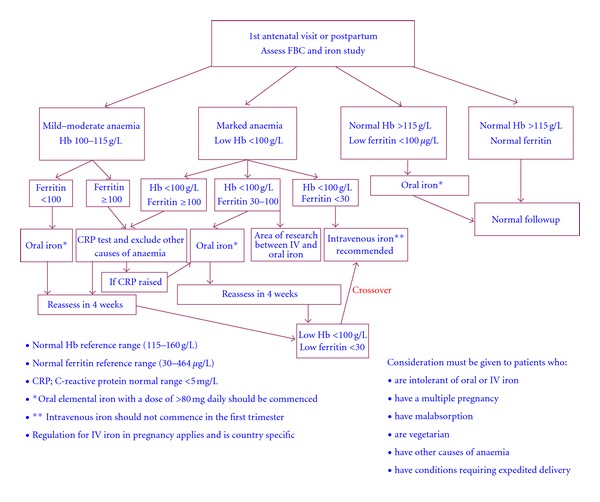
Proposed treatment for anaemia in pregnancy and postpartum period based on different randomized and non-randomized trials [7, 25, 30, 41, 42, 52–55].

The WHO has recognised the problem of IDA in the general population as the most debilitating nutritional deficiency worldwide in the twenty-first century, noting women to be at particularly high risk. Such a problem, if ignored and not addressed properly, can have a devastating effect on entire populations with serious consequences. Therefore, the use of intravenous iron should be considered as an effective, rapid, and safe treatment option in some clinical situations. An algorithm for the treatment of iron deficiency anaemia in pregnancy and postpartum period based on different prospective randomised trials is proposed in Figure 1 [7, 25, 30, 41, 42]. The intravenous iron is increasingly employed to avoid or reduce the demand for blood transfusions or for effective rapid repletion of iron stores. Treatment options for IDA should consider the recently developed intravenous iron formulations, which are considered a milestone in the management of IDA (Figure 1).

Overall, the developing world is most vulnerable, especially the poorest and the least educated populations that are disproportionately affected by iron deficiency, and therefore have the most to gain by eradication of IDA. Furthermore, awareness of the magnitude and scale of the IDA problem during pregnancy and also in the non-pregnant female population will help health practitioners in recognising the most appropriate methods of diagnosis and treatment, which are crucial in overcoming such a devastating health problem. A consensus guideline set by world experts in managing IDA in women and in the general population, incorporating new intravenous iron therapies with a global approach of the health and economy aspects of IDA, should be considered. It is worthwhile considering a universal comprehensive IDA management algorithm that offers different evidence-based treatment options and addresses local conditions. However, developing countries with prevalent IDA often have lack of resources. Therefore, it is crucial to adapt a viable programme with the aim of utilising the local available resources effectively. Perhaps prioritising the treatment of IDA and increasing the awareness among the community of such a chronic devastating problem of paramount importance is the key for success and sustainability of such a programme. Certainly, successful eradication of IDA will result in huge benefits for community health and productivity with a major health saving not only in the developing world but also in developed nations.

## Figures and Tables

**Table 1:** Recently available intravenous (IV) iron preparations.

Name of the IV iron preparation	Status of registration	Indications	Test dose	Duration of infusion	Max dose in single infusion	Reference
^ ∗^Ferumoxytol (Feraheme, AMAG Pharmaceuticals, Inc., USA)	FDA approved	Treatment of iron-deficiency anaemia in adult patients with CKD**	None	1 minute	510 mg	[46, 47]

^∗ ^Ferric carboxymaltose (Ferinject, Vifor Pharma, Glattbrugg, Switzerland)	Approved in Europe,FDA-approval is sought	Treatment of iron-deficiency anaemia in adult patients	None	15 minutes	20 mg/kg with max dose of 1000 mg	[48, 49]

^ ∗ ^Iron isomaltoside (MonoFer, Pharmacosmos A/S, Holbaek, Denmark)	Approved in Europe FDA-approval is sought	Treatment of iron-deficiency anaemia in adult patients with CKD**	None	60 minutes	No max dose given at rate of 20 mg/kg	[50, 51]

FDA, Food and Drug Administration; CKD, chronic kidney disease; Max, maximum. ^ ∗^No available data in pregnancy; however, if the benefit of treatment is judged to outweigh the potential risk to the fetus, the treatment should be confined to second and third trimester of pregnancy. ^ ∗∗^Extended approval is sought.

**Table 2:** Comparison of costs of different oral and IV iron preparations.

Product	Scientific name	Route of	Elemental iron/	Approximate cost
/manufacturer		administration	recommended dose	
Ferro-Liquid (AFT Pharmaceuticals)	Ferrous sulphate	Oral liquid 250 mL (30 mg/5 mL)	90 mg/15 mL/day	$19.35 per bottle Total cost per pregnancy $309

Ferro-Tab (AFT Pharmaceuticals)	Ferrous fumarate200 mg	Oral tablet 60 tabs	67.5 mg/day	$11.62 per package Cost per tablet $0.2 Total cost per pregnancy $54

FGF (Abbott)	Ferrous sulfate 250 mg (+ folic acid)	Oral tablet 30 tabs	80 mg/day	$7 per package Cost per tablet $0.23 Total cost per pregnancy $62

Fefol (Pharmacare Laboratories)	Ferrous sulfate 270 mg (+ folic acid)	Oral tablet 30 tabs	87 mg/day	$9.95 per package Cost per tablet $0.33 Total cost per pregnancy $89

Ferro-F-Tab (AFT Pharmaceuticals)	Ferrous fumarate 310 mg (+ folic acid)	Oral tablet 60 tabs	100 mg/day	$12.79 per package Cost per tablet $0.21 Total cost per pregnancy $57

Ferrograd C (Abbott)	Ferrous sulfate 325 mg	Oral tablet 30 tabs	105 mg/day	$8.16 per package Cost per tablet $0.27 Total cost per pregnancy $73

Ferro-gradumet (Abbott)	Ferrous sulfate 325 mg	Oral tablet 30 tabs	105 mg/day	$6.56 per package Cost per tablet $0.21 Total cost per pregnancy $57

		Intravenous iron		

Ferrum-H (Vifor Pharma Pty Ltd)	Iron polymaltose	IV 100 mg ampule package 5 amps	No maximum (Max) dose at a single administration	$49.57 for 5 × 100 mg

Ferrosig (Sigma Pharmaceuticals)	Iron polymaltose 100 mg ampoule	IV 100 mg ampoule package 5 amps	No max dose at a single administration	$49.57 for 5 × 100 mg

Venofer (Aspen Pharmacare)	Iron sucrose	IV 100 mg ampule package 5 amps	Max single dose 300 mg	$139.48 for 5 × 100 mg

Ferinject (Vifor)	Ferric carboxymaltose	IV 100 mg and 500 mg ampules	Max single dose 1000 mg	100 mg: $35/vial 500 mg: $136/vial

MonoFer, (Pharmacosmos)	Iron isomaltoside	IV 100 mg ampule	No max dose at a single administration	Not available

Feraheme, (AMAG Pharmaceuticals, Inc.)	Ferumoxytol	IV 100 mg ampule	Max single dose 510 mg	Not available

Cost is based on Pharmaceutical Benefit Scheme (PBS) listing price in Australia in AUD which is equivalent to USD (1:1) at the time of analysis. However the prices are only approximate as there is considerable variability depending on purchasing situation or country of origin.
